# Factors Influencing Acceptance of Grasshoppers and Other Insects as Food: A Comparison between Two Cities in Malaysia

**DOI:** 10.3390/foods11203284

**Published:** 2022-10-20

**Authors:** See Meng Lim, Chai Nei Thien, Abdoul Karim Toure, Bee Koon Poh

**Affiliations:** 1 Nutritional Sciences Programme & Centre for Community Health Studies (ReaCH), Faculty of Health Sciences, Universiti Kebangsaan Malaysia, Kuala Lumpur 50300, Malaysia; 2 Faculty of Quranic and Sunnah Studies, Universiti Sains Islam Malaysia, Nilai 71800, Malaysia

**Keywords:** entomophagy, edible insects, preference, barrier

## Abstract

Entomophagy has recently sparked widespread attention worldwide. Although entomophagy is not a foreign dietary practice in Malaysia, acceptance of insects as food among Malaysians is still unclear. This study aimed to determine the acceptance of insects as food and its influencing factors among adults living in Klang Valley (Peninsular Malaysia) and Kuching, Sarawak (East Malaysia). A cross-sectional survey was conducted involving 292 adults from Klang Valley (*n* = 144) and Kuching (*n* = 148). Data was collected through self-administrated online questionnaires. Although most respondents (96.7%) had prior knowledge of people eating insects, only 30.1% of respondents accepted insects as food, and only 18.2% reported that they would be willing to include insects in their daily diet. There was no statistically significant difference in acceptance rates between Klang Valley and Kuching. The main factors influencing respondents’ acceptance of insects as food were insect texture, food safety issues and aversion toward insects. In conclusion, the acceptance of insects as food among adults in Klang Valley and Kuching is still low, with sensory characteristics, food safety and sentiments of distaste being the main barriers. Future studies involving insect tasting and in-depth focus group discussion are needed for deeper insights into the acceptance of insects as food.

## 1. Introduction

Entomophagy is the practice of eating insects, which has been widely practiced in both Western and Asian countries. Insect eating is practiced by over 2 billion people worldwide on a daily basis [1]. According to Tang et al. (2019), about 2000 species of insects are known to be edible, and these insects commonly live in aquatic or terrestrial areas [2]. Among the edible insects, beetles, caterpillars, bees, wasps and ants are the most frequently eaten species worldwide [3]. Previous studies have reported that insects can be eaten raw, cooked or baked [4,5,6]. In addition, insects can be ground into insect powder before mixing with other ingredients to make food products, such as biscuits and bread.

Edible insects are generally nutrient-dense; they contain amounts of different nutrients, such as protein, fat, fibre, vitamins and minerals that are comparable with other animal-source food products [7]. Importantly, edible insects have been recognised as a good alternative for meeting the increasingly high demand for protein sources due to the world’s growing population [8]. In addition, insect farming is more sustainable and environmentally friendly than other protein sources [8]. The livestock farming sector has been identified as one of the main contributors to climate change due to the high emissions of greenhouse and ammonia gases from cattle, swine and poultry. Therefore, the production of insects on a large scale has been suggested as an alternative to meet the growing demand for livestock, which is due to the rapid growth of the world’s population, without having a significant impact on the environment [7].

Recently, studies related to the determination of acceptance and willingness of people to accept or try insects as food are growing immensely in different countries [9,10,11,12]. For example, Wilkinson et al. (2018) reported that about 17.8% of Australians were willing to accept insects as food [12], while another study in Belgium showed that 19.0% of the population were ready to accept insects as a meat substitute [11]. Moreover, past studies have found that Asian and Western countries have different levels of acceptance of insects as food. For instance, the population in China was found to have a higher acceptance of insects as food compared to populations in Western countries such as Germany [13]. One possible reason for this difference is that China has a pre-history of eating insects as one of its cultural practices [13]. Therefore, a person’s level of acceptance of insects as food can be influenced by their living environment and exposure to insect products [14]. Apart from that, several other factors, including gender, age, ethnicity, education level, food neophobia, feelings of disgust, price of the product and the form of insects to be consumed have also been identified as factors that can influence the acceptance of insects as food [15,16,17].

Malaysia is a country located in Southeast Asia. It is composed of two non-contiguous regions: Peninsular Malaysia, which is also called West Malaysia, and East Malaysia, which is on Borneo Island. Klang Valley, the central part of the west coast of Peninsular Malaysia, is one of the most developed regions in Malaysia. It consists of the Federal Territory of Kuala Lumpur (the national capital of Malaysia) and its few adjoining cities and towns in the state of Selangor, including Gombak, Hulu Langat, Klang and Petaling [18]. On the other hand, East Malaysia consists of two states, Sabah and Sarawak, and the Federal Territory of Labuan. The largest city in East Malaysia is Kuching, which is also the capital of Sarawak. In terms of religion, the majority of the population in the Klang Valley are Muslims (46.4%), followed by Buddhists (35.7%), Hindus (8.5%) and Christians (5.8%). The majority religion in Sarawak, including Kuching, is Christianity (42.6%), followed by Islam (31.3%), Buddhism (12%) and tribal beliefs (5.2%) [19].

In Malaysia, the practice of eating insects is one of its food cultures, more commonly practiced among people living in inland areas such as the *Bumiputera* (indigenous people) in Sabah and Sarawak [20]. However, no published studies are found related to the acceptance of and willingness to eat insects as food among the general Malaysian population. The factors influencing the acceptance of insects as food among Malaysians and whether the level of acceptance differs between Peninsular (West) Malaysia and East Malaysia are also unclear. Since Islam is the official religion in Malaysia, Muslims will consume foods that are regarded as permissible (*halal*) and will not consume any forbidden foods and beverages (*haram*) [21,22]. Grasshopper is recognised as a halal food that can be eaten by Muslims, and this has been agreed upon by scholars from all four Sunni madhhabs (i.e., schools of thought within Fiqh or Islamic jurisprudence), including Hanafi, Maliki, Shafi’i and Hanbali [23]. Therefore, this study aimed to determine the acceptance of grasshoppers and other insects as food among adults who had been born and raised in Klang Valley (Peninsular Malaysia) or Kuching, Sarawak (East Malaysia), as well as among those who had lived there for more than five years. In addition, we also aimed to identify factors influencing their acceptance, which either motivate or discourage them from consuming grasshoppers and other insects as food.

## 2. Materials and Methods

### 2.1. Study Design and Respondents

This cross-sectional survey was conducted online using Google Forms, between August and October 2021, among Malaysian adults aged 18 years and above. The link to the Google Forms of the study was disseminated through social media, including WhatsApp, Facebook, Instagram, Telegram and LinkedIn, as well as through the respondents’ networking (snowball sampling method). Respondents who were born and raised in Klang Valley or Kuching, Sarawak and/or had lived there for more than five years were eligible to participate in this study. Both study locations were chosen because they have the highest estimated population in Peninsular and East Malaysia [24]. The sample size was calculated using the Krejcie and Morgan (1970) formula [25], with the desired confidence level of 95%, level of precision of 0.05, population proportion of 0.19 on acceptance of insects as meat substitute [11] and the 2020 population size of 8,418,583 (Klang Valley: 7,715,883; Kuching: 702,700) according to the Department of Statistics Malaysia [26,27,28]. After taking into account a 20% non-response rate, the total number of subjects needed for the study was 296.

### 2.2. Ethics and Consent

The study was conducted in agreement with the Declaration of Helsinki, and the protocol was approved by the Research Ethics Committee of the Universiti Kebangsaan Malaysia (JEP-UKM-2021-482). Participants were provided information on the study background, objectives and scope of questions, and they were required to provide their consent before answering the survey. They were able to withdraw from the survey at any time without providing a reason.

### 2.3. Questionnaire

The questionnaire used in this study was adapted from questionnaires of previous studies, with permission [12,29]. The questionnaire was prepared in English and Malay languages. Prior to data collection, a pre-test was conducted to ensure clarity and ease of understanding of the questionnaire by the respondents. The self-administered questionnaire for the study consisted of two parts (Figure S1). The first part covered the sociodemographic information of the respondent, such as gender, age, religion, education level, personal income and household income. The following part focused on the respondent’s acceptance and the factors that influenced their acceptance. In this part, there were two different sets of the same group of questions, in which the type of edible insect was specifically referred to as grasshoppers for Muslim respondents in one set, whereas the other set consisted of many types of edible insects, including grasshoppers, sago worms, ants, crickets and larvae, for non-Muslim respondents. Briefly, the second part of the questionnaire consisted of questions related to the respondent’s experience eating grasshoppers and other insects, which were determined through questions such as “Have you heard of people eating grasshopper/insect before?” and “Have you ever consumed grasshopper/insect previously?” Next, questions such as “Will you accept eating grasshopper/insect as food” and “Are you willing to eat grasshopper/insect as food in your daily life?” were also asked to determine the willingness to accept grasshoppers or insects as food. In addition, respondents’ acceptance based on the insect’s appearance was also determined, where subjects were asked to choose how likely they would be to accept roasted grasshopper/insect, chocolate-coated grasshopper/insect, a cooked meal containing grasshopper/insect and biscuits made with grasshopper/insect flour using a 5-point Likert Scale starting with “1 = Very unlikely” to “5 = Very likely”. A question related to the factors that influence the acceptance of grasshoppers or insects as food was also asked. A total of nine factors, including the feeling of disgust, taste, insect texture, food safety issues, nutritional value, price, peer influence, the role of parents or family members and the availability of grasshoppers or insects, were asked in the questionnaire. An open-ended question was also included to understand if any other factors may also influence the respondents’ acceptance of grasshoppers or insects as food. In addition, respondents were asked to what extent the practice of insect-eating could address specific issues, such as nutrition, food security, environmental sustainability, limited sources of agricultural land, animal welfare and high demand for protein sources, using a 5-point Likert Scale starting with “1 = Strongly disagree” to “5 = Strongly agree”. The last question in the questionnaire was related to the reasonable price that respondents would spend on purchasing grasshoppers or other insects or related food products.

### 2.4. Data Analysis

Data were analysed using IBM SPSS Statistics version 25 (Chicago, IL, USA). Descriptive statistics are presented as frequency and percentage for categorical variables as well as mean and standard deviation for continuous data. The Friedman test followed by a Wilcoxon signed-rank post hoc test was used to determine the main factors influencing the acceptance of insects as food. In addition, the chi-square test of independence, or Fisher’s exact test if ≤20% of expected cell counts were <5, was used to compare the acceptance of insects as food between the Klang Valley and Kuching respondents. The statistical significance level was set at *p* < 0.05 for all tests.

## 3. Results

### 3.1. Sociodemographic Characteristics of the Respondents

The sociodemographic characteristics of the respondents, such as gender, age group, race and religion, according to the study location, are shown in Table 1. A total of 144 subjects from Klang Valley (49.3%) and 148 subjects from Kuching (50.7%) were recruited for this study. The majority of the respondents were female (68.2%), between the ages of 18–29 years old (78.4%) with a median age of 23 years, and were non-Muslim (77.4%). Half of the study respondents had attained the secondary school education level (50.7%). In addition, about half of the respondents had a personal income of RM1799 or below (~USD401 and below; 57.5%), with most respondents having a low to moderate monthly household income (70.2%). There were significant differences (*p* < 0.01) in all sociodemographic characteristics, except gender, between respondents from Kuching and Klang Valley.

### 3.2. Insect-Eating Practices: Awareness and Experience of Respondents

Table 2 shows the awareness and experience of eating grasshoppers or other insects among the respondents by study location and religion. Most respondents reported that they had heard of people eating grasshoppers or other insects (96.7%). The three main channels through which the respondents learnt about entomophagy were the internet (79.0%), social media (76.6%) and television (73.4%) (Figure S2). However, despite knowing about the practice of eating insects, only 6 Muslim respondents (9.1%) stated that they had eaten grasshoppers, and only 98 non-Muslim respondents (43.4%) had eaten insects previously. Among all the insects, sago worm (48.5%), cricket (36.9%), grasshopper (31.1%), ant (24.3%) and larvae (22.3%) were the most frequently consumed by non-Muslim respondents (Figure S3).

### 3.3. Acceptance of Grasshoppers and Other Insects as Food

The acceptance of grasshoppers and other insects as food based on location and religion is shown in Table 3. The overall acceptance of the respondents was low, with only 88 respondents (30.1%) reporting that they would accept grasshoppers or other insects as food. Only 53 respondents (18.2%) reported that they would be willing to eat grasshoppers or other insects as part of their daily diet. However, we found no significant differences in the acceptance rates of grasshoppers and other insects as food from respondents between Klang Valley and Kuching. Similarly, there were also no significant differences between Muslim and non-Muslim respondents regarding the acceptance of grasshoppers or other insects as food, although the acceptance by Muslim respondents was slightly lower compared to non-Muslim respondents.

The acceptance of respondents toward eating grasshoppers and other insects as food based on sociodemographic and socioeconomic characteristics was also determined (Tables S1 and S2). Overall, the current study found that the acceptance by males was significantly higher (*p* < 0.01) compared to females. Those in the younger age group (between 18 and 39 years old) showed higher acceptance of eating grasshoppers or other insects as food. Besides, respondents with higher personal income were more likely to accept grasshoppers or other insects as food daily. The current study also discovered that respondents with higher monthly household incomes were more readily accepting eating grasshoppers or other insects as food. With regards to the location of the respondents, the acceptance of males was significantly higher (*p* < 0.01) compared to females only in Klang Valley, with no significant difference between the sexes in Kuching. However, based on income groups, only respondents in Kuching showed significant differences (*p* < 0.05), with a higher personal income and higher monthly household income groups being more accepting of grasshoppers and other insects as food in daily life; there was no such difference found in Klang Valley.

In terms of the visual acceptability of grasshoppers and other insects as food, biscuits made with grasshopper or insect flour received the highest acceptance compared to other forms (*p* < 0.001). However, no significant difference was found between the acceptance of roasted grasshopper or insect and biscuits made with grasshopper or insect flour. In addition, respondents in this study reported that they were less likely to accept grasshoppers or other insects coated with chocolate than cooked meals containing grasshoppers or other insects (Table S3). No significant differences were found for respondents from both the Klang Valley and Kuching in terms of the visual acceptability of grasshoppers and other insects as food.

### 3.4. Factors Affecting Respondents’ Acceptance of Grasshoppers or Other Insects as Food

Overall, the present study shows that insect texture, food safety issues and feelings of disgust were the three most important barriers to the acceptance of insects as food, with no significant differences between their mean rank scores (Table 4). Other factors that may also influence the acceptance of grasshoppers and other insects as food include taste, nutritional value, price, the role of parents or family members, peer influence and availability. Moreover, the main factors that influenced respondents’ acceptance of each study location were also determined. It was found that the top three mean rank scores were the same for Klang Valley and Kuching respondents, which were insect texture, feeling of disgust and food safety issues. Moreover, the factors that influence the acceptance of grasshoppers or other insects as food among Muslim and non-Muslim respondents were also determined in this study (Table 4). Interestingly, the main factors influencing the acceptance of grasshoppers as food for Muslim respondents and grasshoppers and other insects for non-Muslim respondents were the same, where insect texture, food safety issues and feelings of disgust were the top three barriers. In addition to the nine listed options in the questionnaire, some respondents also stated other factors that may lower their acceptance of grasshoppers and other insects as food, such as feelings of fear and weirdness, cultural and religious aspects and lack of knowledge about insect-eating.

Table 5 shows the issues that could be addressed through the practice of insect-eating according to the respondents’ locations and religions. Overall, the respondents agreed that eating insects could help to mitigate the high demand for protein sources with the highest mean rank score (3.76), and it had no significant differences with other issues, including nutrition, food security, environmental sustainability and limited demand for protein sources. Similarly, respondents from Klang Valley and Kuching, as well as those of Muslim faith, agreed that eating insects can help to meet the high demand for protein sources. For non-Muslim respondents, they agreed that nutrition is the main issue that can be solved by eating insects, without any significant difference compared to the high demand for protein sources as the issue.

Lastly, more than half of the respondents (60%) stated that the reasonable price for 100-g grasshoppers or other insects should be below RM9.99 (~USD2.23), and another 36.3% of the respondents chose RM10.00-RM29.99 (~USD2.23–USD6.69) as the reasonable price (Figure S4).

## 4. Discussion

The practice of consuming insects, also known as “entomophagy”, has recently gained immense attention. Edible insects are commonly promoted as an alternative source of protein due to their high nutritional value, low environmental impact, high availability and shorter life cycles. Although most of the adults in this survey have heard of people eating grasshoppers or other insects in the past, neither Klang Valley nor Kuching adults were particularly eager or interested in doing so in their daily diet. Furthermore, the three factors that influenced adults’ acceptance of grasshoppers or other insects as food in this study were the insect texture, concerns about food safety and sentiments of distaste.

The findings of this study on Malaysians’ willingness to accept eating grasshoppers or other insects as food are congruent with those of an earlier study, which found that Australia’s population had a low propensity to do so [12]. Additionally, the majority of the residents in Poland also stated that they would not consume insects as food on a regular basis [10]. As a result, even while interest in eating insects as food is growing, the general public still has a low level of acceptance for the practice. In this study, we discovered that non-Muslims were more accepting of practicing insect-eating than Muslims. The cultural and religious distinctions between Muslim and non-Muslim respondents may be one factor. Muslims are frequently more cautious with food selection, especially when it comes to novel or unfamiliar foods, the ingredients in the food product and how the food business handles the food [30]. Additionally, the Islamic laws, which stipulate that Muslims may only consume food that has been certified as *halal*, are a major factor in the Muslim community’s willingness and acceptance of entomophagy [23].

The visual appearance of a particular food can affect a person’s food choice [31,32]. It was found that respondents in this study commonly preferred processed insects or invisible forms of insects, such as biscuits made with grasshoppers or other insect flour. Past studies have also demonstrated that the powder form of insect products is better accepted than other forms [33,34]. Interestingly, while we found that the acceptance of processed, invisible form of grasshoppers or other insects was the highest, there was no significant difference when it was compared to the unprocessed, visible form of grasshoppers or insects (roasted grasshoppers or other insects). Possibly due to Malaysia’s tropical climate, where the majority of people are considerably more likely to come into contact with uncooked or raw insects, the acceptance of either invisible or visible insects among Kuching and Klang Valley adults was similar. The results of this study also demonstrate that grasshoppers or other insects coated with chocolate were the least preferred form, suggesting that consumers prefer insects blended into savoury products over sweetened products, similar to the findings in previous studies on Italian or Dutch consumers [35,36]. Therefore, the incorporation of insects into sweetened products, such as desserts, chocolate and cakes, is considered less undesirable by consumers [35], probably because insects are generally classified as protein substitutes [37].

An individual’s sociodemographic background, including their sex, age, education and financial background, also affects their level of acceptance of edible insects. The current study, which found that the male respondents’ acceptance of entomophagy was higher than their female counterparts, is in line with past studies [10,36,38]. In relation to that, Menozzi et al. (2017) also reported that the male consumers’ acceptance of food products containing insect flour was significantly higher compared to female consumers [39]. This phenomenon may be a result of their personalities, since males are prone to be more adventurous than females when trying new meals, including eating insects [12]. As reported previously, females also have a higher fear and disgust toward different arthropods [40]. In this study, it was discovered that respondents who were younger in age accepted insects as meals more readily. This finding is consistent with the study done by Castro and Chambers IV (2019), which also revealed that the younger age group (18 to 34 years old) was more inclined to eat an insect-based diet [41]. Therefore, younger consumers are generally more likely to accept new types of food, including having a more positive attitude toward the practice of insect-eating [42]. In addition, the acceptance of eating insects has been shown to correlate positively with education [43]. A previous study, which reported that the acceptance of eating insects as food was higher among postgraduates, is similar to the findings obtained in the current study, where higher acceptance was found in those who had attained tertiary education level [44]. Higher-educated people typically have a better understanding of entomophagy, which leads to a more open mindset toward experimenting with new foods [44]. Moreover, other research has examined how wealth level affects people’s willingness to eat insects [45,46]. Similar to the results of the current study, those with higher personal incomes tend to accept entomophagy more readily than people who earn less money [10].

In this study, insect texture, food safety concerns and revulsion were the key factors in respondents’ acceptance of insects as food. Our results are consistent with earlier studies. For instance, Mancini et al. (2019) revealed that one of the key factors affecting consumer acceptability of insects as food is insect texture [14]. Tan et al. (2017) demonstrated how differing insect textures would affect customer acceptance and desire to buy insect-based products in the Netherlands, providing additional support for this finding [47]. Additionally, Roma et al. (2020) discovered that food safety is a factor that will affect consumers’ acceptance in Italy [48]. Jensen and Lieberoth (2019) also found that consumers often associate their food choices with food safety issues, particularly disease and contamination [33]. Houbraken et al. (2016) also found that contamination happens when mealworms hold on to a lot of pesticides in their bodies [49]. Since insects have a high potential to store and accumulate parasites in their bodies that can later cause infection when eaten by humans [5,50], the safety aspect should be one of the priorities in encouraging the practice of eating insects. Apart from that, Lammers et al. (2019) also found that revulsion significantly influences consumer acceptance of burgers containing insects and buffalo worms in Germany [34]. Chang et al. (2019)’s findings about how people in Taiwan feel about eating insects [51] and Bae and Choi (2021)’s findings from Korea [52] were similar to the results of this study. Both studies found that revulsion is the main reason why people do not purchase or eat insects. Therefore, it is clearly shown that sentiments of distaste will influence one’s acceptance of edible insects in either Western or Eastern society.

In this study, adults from Kuching and Klang Valley rated that the high demand for protein sources and nutritional problems could be overcome by eating insects. The rapid growth of the world population has indirectly increased the demand for food, especially protein sources [53]. The growth rate of insects is faster than that of other animals, allowing more production to meet the high demand for protein sources and reduce hunger and food insecurity around the world [2]. Therefore, eating insects, which serve as an alternative source of protein, is an effective strategy that can address this problem [1,54]. In relation to that, insects also have a higher nutritional content than conventional species, such as cattle and poultry [7]. A previous study also reported high antioxidant activities in edible insects, such as mulberry silkworms [55]. Additionally, previous studies suggested that insect farming has minimal impact on the environment because greenhouse gas emissions were lower than those of other livestock [56,57]. The small body size of insects allows for vertical farming, which can reduce agricultural land use [58]. Although there is no explicit legislation in Malaysia to control the insect industry [59], the benefits of entomophagy for environmental sustainability may offer a huge opportunity for entrepreneurship in the country.

This study has several limitations that should be acknowledged. Firstly, this study used convenience and snowball sampling to collect data. Only adults from Kuching and Klang Valley participated in this study, so the results cannot be generalised to all Malaysians. Despite this, to the best of our knowledge, this is the first study in Malaysia to compare adults from Kuching and Klang Valley’s acceptance of grasshoppers and other insects as food and its influencing factors. In addition, the data from this study can be used as a reference for future studies and ministries such as the Ministry of Health and the Ministry of Plantation Industries and Commodities in developing appropriate and effective interventions to promote insect-eating in Malaysia. Moreover, this study only used a questionnaire as the survey tool to assess the acceptance of grasshoppers and insects as food. Future studies can include tastings of insects and in-depth focus group discussions to more accurately ascertain if the population will accept entomophagy. It would be especially important to determine whether there are any differences in the acceptance of insects as food between adults who live in urban areas and those who live in rural regions, as well as to understand their perspectives on the importance of entomophagy. Additionally, it would also be worthwhile to investigate the source of insects if they have been practising entomophagy.

## 5. Conclusions

Even though Malaysia has a history of insect-eating and most Malaysians have prior knowledge of people eating insects, the acceptance of grasshoppers and other insects as food among adults in Kuching and Klang Valley is generally still low. While the geographical location seems to not influence the acceptance rate, the type of insects to be consumed is an important issue to be taken into consideration, as nearly two-thirds of Malaysia’s population are Muslims. Insect texture, food safety issues and sentiments of distaste are the main factors influencing the lack of acceptance of grasshoppers and other insects as food. These results can be utilised by the industry to guide manufacturing and marketing strategies, such as which insects can be added and the information included in the food packaging, to increase consumers’ acceptance. Future studies might include insect tasting sessions or in-depth focus group discussions in order to more accurately assess how people feel about the practice of eating insects in Malaysia.

## Figures and Tables

**Table 1:** Sociodemographic characteristics of the respondents according to the study location, *n* (%).

	All (*n* = 292)	Klang Valley (*n* = 144)	Kuching (*n* = 148)	*p* Value
**Gender**				0.431
Male	93 (31.8)	49 (34.0)	44 (29.7)	
Female	199 (68.2)	95 (66.0)	104 (70.3)	
**Age group**				*p* < 0.001 ^†††^
18–29 years old	229 (78.4)	99 (68.8)	130 (87.8)	
30–39 years old	37 (12.7)	32 (22.2)	5 (3.4)	
40–49 years old	18 (6.2)	11 (7.6)	7 (4.7)	
50 years old and above	8 (2.7)	2 (1.4)	6 (4.1)	
**Religion**				*p* < 0.001 ^†††^
Muslim	66 (22.6)	56 (38.9)	10 (6.8)	
Non-Muslim	226 (77.4)	88 (61.1)	138 (93.2)	
**Highest education level**				0.002 **
Primary	1 (0.3)	0 (0.0)	1 (0.7)	
Secondary	148 (50.7)	60 (41.7)	88 (59.5)	
Tertiary	143 (49.0)	84 (58.3)	59 (39.9)	
**Occupation**				0.003 **
Government/Semi-government servant	24 (8.2)	15 (10.4)	9 (6.1)	
Private worker	70 (24.0)	45 (31.3)	25 (16.9)	
Self-employed	12 (4.1)	6 (4.2)	6 (4.1)	
Unpaid worker/Unemployed/Retired	25 (8.6)	15 (10.4)	10 (6.8)	
Students	161 (55.1)	63 (43.8)	98 (66.2)	
**Personal income**				*p* < 0.001 ^†††^
RM 1799 and below ^‡^	168 (57.5)	68 (47.2)	100 (67.6)	
RM 1800—RM 2600	21 (7.2)	8 (5.6)	13 (8.8)	
RM 2601—RM 5499	48 (16.4)	35 (24.3)	13 (8.8)	
RM 5500 and above	26 (8.9)	21 (14.6)	5 (3.4)	
No response	29 (9.9)	12 (8.3)	17 (11.5)	
**Household income ^‡‡^**				*p* < 0.001 ^†††^
Less than RM 4860 (B40 group)	94 (32.2)	30 (20.8)	64 (43.2)	
RM 4860—RM 10959 (M40 group)	111 (38.0)	63 (43.8)	48 (32.4)	
More than RM 10960 (T20 group)	40 (13.7)	30 (20.8)	10 (6.8)	
No response	47 (16.1)	21 (14.6)	26 (17.6)	

^‡^ RM, Malaysian Ringgit; 1USD = RM 4.481 as of 29 August 2022. ^‡‡^ Source: Household Income and Basic Amenities Survey Report 2019, Department of Statistics Malaysia. Significant differences between two study locations using the Fisher Exact test: ** *p* < 0.01. Significant differences between two study locations using the Chi-square test: ^†††^ *p* < 0.001.

**Table 2:** Awareness and experience of eating grasshoppers or other insects as food, *n* (%).

Question	All (*n* = 292)	Location		Religion	
		Klang Valley (*n* = 144)	Kuching (*n* = 148)	Muslim (*n* = 66)	Non-Muslim (*n* = 226)
1. Have you heard of people eating grasshoppers/insects before?					
Yes	286 (96.7)	141 (97.9)	145 (98.0)	63 (95.5)	223 (98.7)
No	6 (3.3)	3 (2.1)	3 (2.0)	3 (4.5)	3 (1.3)
2. Have you ever consumed grasshoppers/insects previously?					
Yes	104 (35.6)	46 (31.9)	58 (39.2)	6 (9.1)	98 (43.4)
No	188 (64.4)	98 (68.1)	90 (60.8)	60 (90.9)	128 (56.6)

**Table 3:** Acceptance of grasshoppers and other insects as food based on location and religion, *n* (%).

	Will You Accept Eating Grasshopper/Insects as Food			Are You Willing to Eat Grasshopper/Insect as Food in Your Daily Life?		
	Yes	No	*p*-Value	Yes	No	*p*-Value
**All subjects (*n* = 292)**	88 (30.1)	204 (69.9)		53 (18.2)	239 (81.8)	
**Location**			0.358			0.793
Klang Valley (*n* = 144)	47 (32.6)	97 (67.4)		27 (18.8)	117 (81.2) 122	
Kuching (*n* = 148)	41 (27.7)	107 (72.3)		26 (17.6)	(82.4)	
**Religion**			0.564			0.279
Muslim (*n* = 66)	18 (27.3)	48 (72.7)		9 (13.6)	57 (86.4)	
Non-Muslim (*n* = 226)	70 (31.0)	156 (69.0)		44 (19.5)	182 (80.5)	

No significant difference using Chi-square test.

**Table 4:** Factors influencing the acceptance of grasshoppers and other insects as food according to study location and religion.

	Mean Rank				
		Location		Religion	
	All (n = 292)	Klang Valley (n = 144)	Kuching (n = 148)	Muslim (n = 66)	Non-Muslim (n = 226)
**Factors ^1^**					
Insect texture	6.01 ^a^	6.20 ^a^	5.83 ^a^	5.94 ^a^	6.04 ^a^
Food safety issue	6.01 ^ab^	5.80 ^ab^	6.22 ^a^	5.79 ^ab^	6.08 ^a^
Disgust	5.96 ^ab^	5.92 ^ab^	5.99 ^a^	5.69 ^abc^	6.04 ^a^
Taste	5.70 ^b^	5.69 ^b^	5.71 ^a^	5.45 ^abc^	5.77 ^ab^
Nutritional value	4.77 ^c^	4.67 ^c^	4.88 ^b^	4.75 ^cd^	4.78 ^c^
Availability	4.32 ^cd^	4.60 ^c^	4.04 ^c^	4.77 ^bcd^	4.19 ^d^
Price	4.09 ^d^	4.09 ^c^	4.04 ^c^	3.77 ^d^	4.19 ^d^
Role of parents or family members	4.08 ^d^	3.96 ^c^	4.20 ^bc^	4.35 ^d^	4.00 ^d^
Peer influence	4.05 ^d^	4.07 ^c^	4.03 ^c^	4.50 ^d^	3.92 ^d^

^1^ Mean rank was reported. ^a,b,c,d^ Different letters within a column indicate significant differences (*p* < 0.05) using the Friedman test and Post Hoc Wilcoxon signed-rank test.

**Table 5:** Issues that could be addressed through entomophagy according to study location and religion.

	Mean Rank				
		Location		Religion	
	All (n = 292)	Klang Valley (n = 144)	Kuching (n = 148)	Muslim (n = 66)	Non-Muslim (n = 226)
**Issues ^1^**					
High demand for protein sources	3.76 ^a^	3.71 ^a^	3.81 ^a^	3.83 ^a^	3.74 ^a^
Nutrition	3.69 ^a^	3.69 ^a^	3.69 ^a^	3.47 ^a^	3.76 ^a^
Environmental sustainability	3.58 ^a^	3.62 ^a^	3.55 ^a^	3.45 ^a^	3.62 ^a^
Limited availability of agricultural land	3.56 ^a^	3.67 ^a^	3.46 ^a^	3.73 ^a^	3.51 ^a^
Food security	3.37 ^a^	3.27 ^a^	3.47 ^a^	3.21 ^a^	3.42 ^a^
Animal welfare	3.03 ^b^	3.03 ^b^	3.02 ^b^	3.31 ^a^	2.95 ^b^

^1^ Mean rank was reported. ^a,b^ Different letters within a column indicate significant differences (*p* < 0.05) using the Friedman test and Post Hoc Wilcoxon signed-rank test.

## Data Availability

Data is contained within the article and supplementary materials.

## Supplementary Materials

The following supporting information can be downloaded at: https://www.mdpi.com/article/10.3390/foods11203284/s1, Figure S1: The schematic drawing of the questionnaire used in the study; Figure S2: Type of channels that the respondents get to know about entomophagy; Figure S3: Type of insects consumed by non-Muslim respondents; Figure S4: Reasonable price for 100 g of grasshopper/insects; Table S1: Acceptance of grasshoppers/other insects as food based on sociodemographic and socioeconomic characteristics; Table S2: Willingness to eat grasshoppers/other insects as food in daily life based on sociodemographic and socioeconomic characteristics; Table S3: Visual acceptability of respondents based on study location.
