# Supplementary material for: Factors Influencing Acceptance of Grasshoppers and Other Insects as Food: A Comparison between Two Cities in Malaysia
Source: Foods. 2022 Oct 20;11(20):3284. doi: 10.3390/foods11203284 (PMC9601470; doi:10.3390/foods11203284)
Supplement: Supplementary file 1 [file foods-11-03284-s001.zip › foods-1920045-supplementary.pdf]

Supplementary data

Figure S1: The schematic drawing of the questionnaire used in the study.

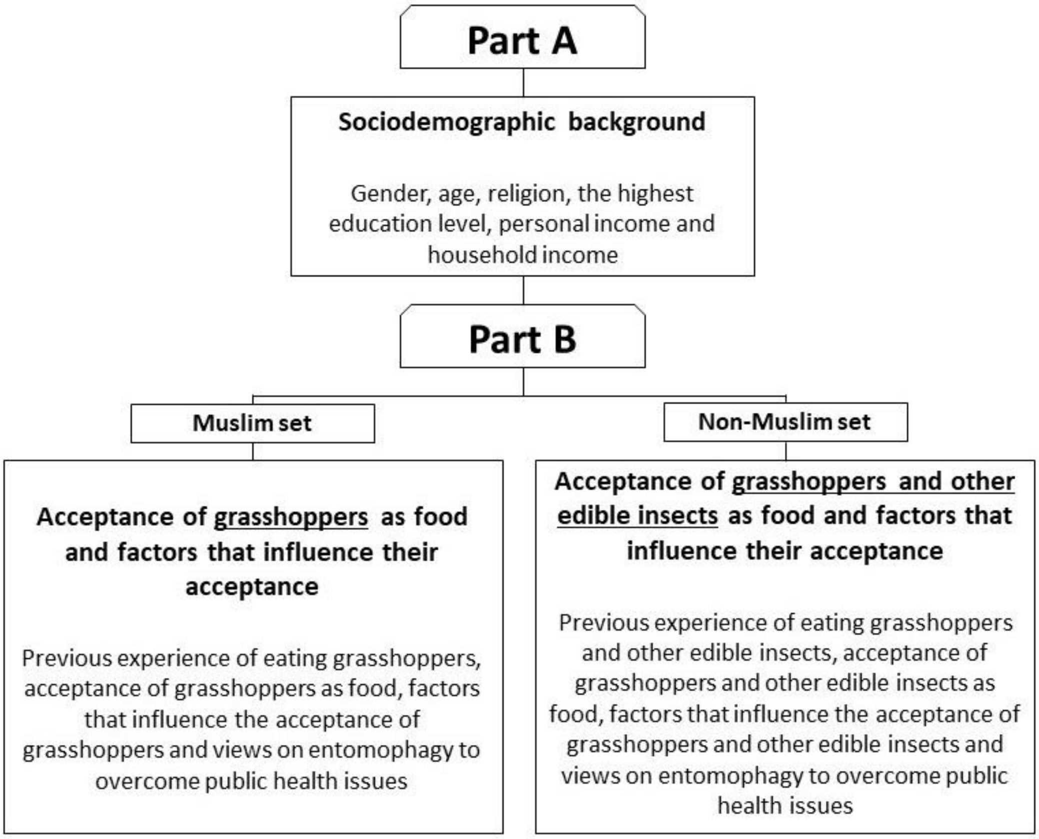

Figure S2: Type of channels that the respondents get to know about entomophagy

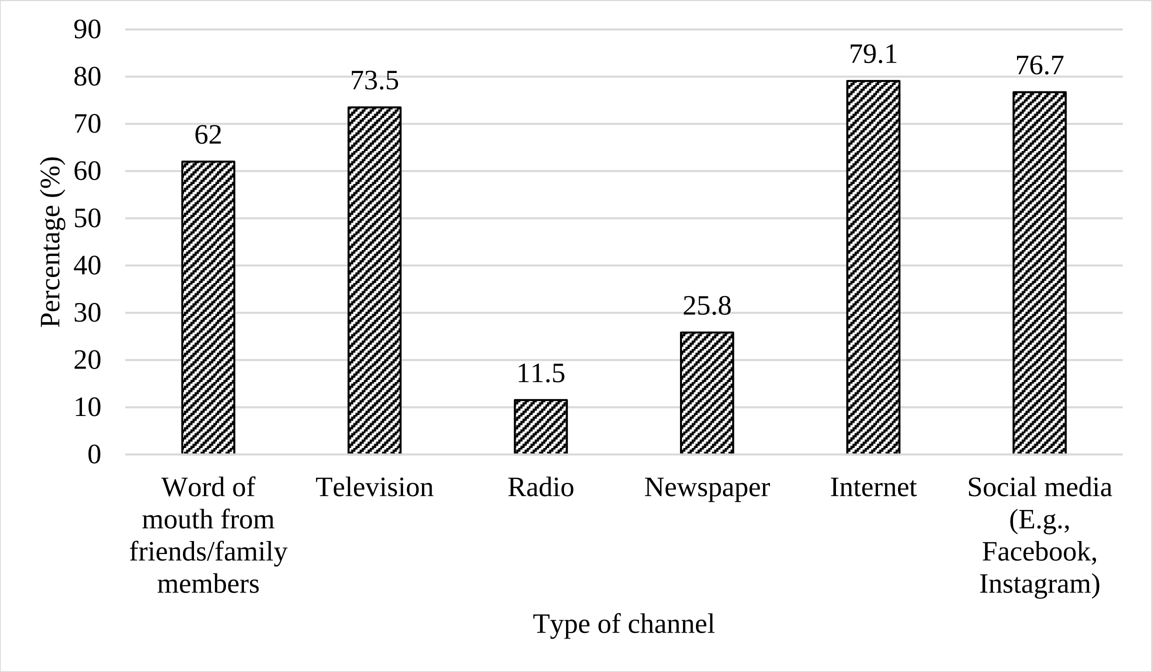

Figure S3: Type of insects consumed by non-Muslim respondents

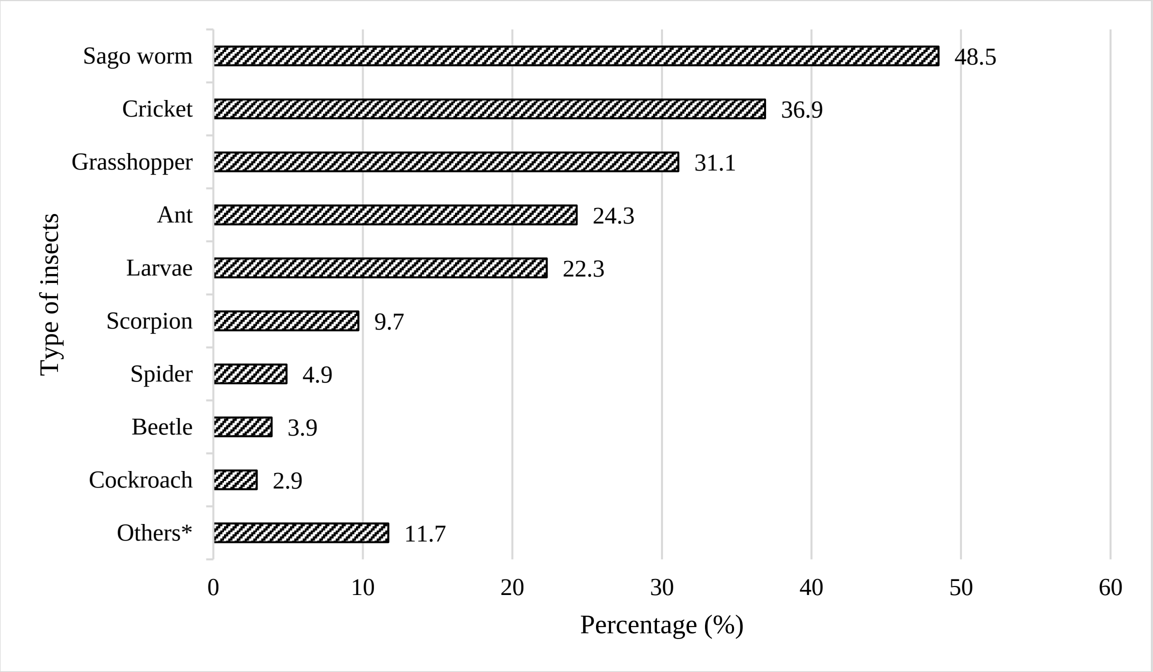

*Mealworm, Silkworm, Bee and Black Soldier Fly

Figure S4: Reasonable price for 100g of grasshopper/insects

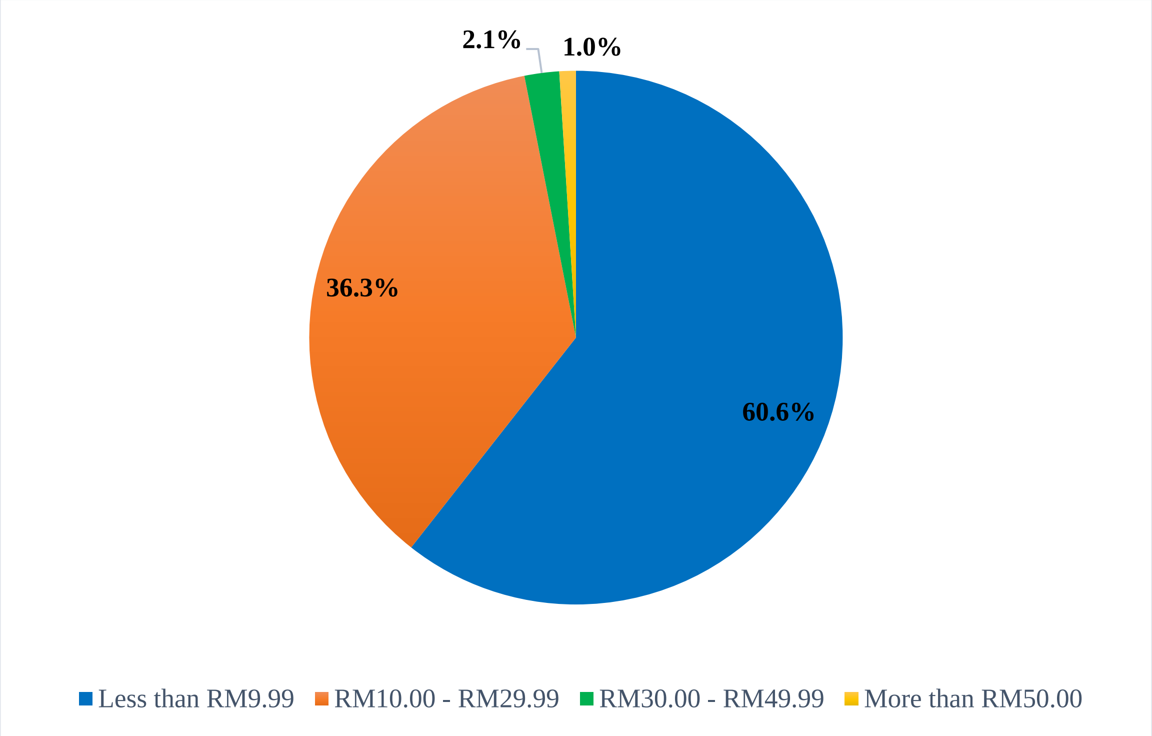

*RM, Malaysian Ringgit; 1USD = RM 4.481 as of 29 August 2022

Table S1: Acceptance of grasshoppers/other insects as food based on sociodemographic and socioeconomic characteristics

Sociodemographic Characterises	**Will you accept eating grasshoppers/insects as food? n (%)**								
	**All (n=292)**			**Klang Valley (n=144)**			**Kuching (n=148)**		
	**Yes**	**No**	***p* value**	**Yes**	**No**	***p* value**	**Yes**	**No**	***p* value**
**Sex**									
Male	42 (45.2)	51 (54.8)	p<0.001	26 (53.1)	23 (46.9)	p<0.001	16 (36.4)	28 (63.6)	0.126
Female	46 (23.1)	153 (76.9)		21 (22.1)	74 (77.9)		25 (24.0)	79 (76.0)	
**Age group**									
18 – 29 years old	61 (26.6)	168 (73.4)		27 (27.3)	72 (72.7)		34 (26.2)	96 (73.8)	
30 – 39 years old	18 (48.6)	19 (51.4)		15 (46.9)	17 (53.1)		3 (60.0)	2 (40.0)	
40 – 49 years old	7(38.9)	11(61.1)	0.042	4 (36.4)	7 (63.6)	0.140	3 (42.9)	4 (57.1)	0.244
50 years old and above	2 (40.0)	6 (75.0)		1 (50.0)	1 (50.0)		1 (16.7)	5 (83.3)	
**Religion**									
Muslim	18 (27.3)	48 (72.7)	0.564	18 (32.1)	38 (67.9)	0.919	0 (0.0)	10 (100.0)	0.062
Non-Muslim	70 (31.0)	156 (69.0)		29 (33.0)	59 (67.0)		41 (29.7)	97 (70.3)	

To be continue…

…continue

Sociodemographic Characterises	Will you accept eating grasshoppers/insects as food? n (%)								
	**All (n=292)**			**Klang Valley (n=144)**			**Kuching (n=148)**		
	**Yes**	**No**	***p* value**	**Yes**	**No**	***p* value**	**Yes**	**No**	***p* value**
**Highest education level**									
Primary	0(0.0)	1(100.0)		0(0.0)	0(0.0)		0(0.0)	1(100.0)	
Secondary	41(27.7)	107(72.3)	0.562	18(30.0)	42(70.0)	0.568	23(26.1)	65(73.9)	0.695
Tertiary	47(32.9)	96(67.1)		29(34.5)	55(65.5)		18(30.5)	41(69.5)	
**Occupation**									
Government/Semi-government servant	11 (45.8)	13 (54.2)		8 (53.3)	7 (46.7)		3 (33.3)	6 (66.7)	
Private worker	29 (41.4)	41 (58.6)		20 (44.4)	25 (55.6)		9 (36.0)	16 (64.0)	
Self-employed	3 (25.0)	9 (75.0)	0.014	1 (16.7)	5 (83.3)	0.046	2 (33.3)	4 (66.7)	0.221
Unpaid worker/ Unemployed/Retired	3 (12.0)	22 (88.0)		3 (20.0)	12 (80.0)		0 (0.0)	10 (100.0)	
Students	42 (26.1)	119 (73.9)		15 (23.8)	48 (76.2)		27 (27.6)	71 (72.4)	

To be continue…

…continue

Sociodemographic Characterises	Will you accept eating grasshoppers/insects as food? n (%)								
	**All (n=292)**			**Klang Valley (n=144)**			**Kuching (n=148)**		
	**Yes**	**No**	***p* value**	**Yes**	**No**	***p* value**	**Yes**	**No**	***p* value**
**Personal income[1]**									
RM 1799 and below*	41 (24.4)	127 (75.6)		15 (22.1)	53 (77.9)		26 (26.0)	74 (74.0)	
RM 1800 – RM 2600	7 (33.3)	14 (66.7)	0.010	4 (50.0)	4 (50.0)	0.052	3 (23.1)	10 (76.9)	0.060
RM 2601 – RM 5499	23 (47.9)	25 (52.1)		15 (42.9)	20 (57.1)		8 (61.5)	5 (38.5)	
RM 5500 and above	11 (42.3)	15 (57.7)		9 (42.9)	12 (67.4)		2 (40.0)	3 (60.0)	
**Household income**[2]**									
Less than RM 4860 (B40 group)	18 (19.1)	76 (80.9)		6 (20.0)	24 (80.0)		12 (18.8)	52 (81.3)	
RM 4860 – RM 10959 (M40 group)	36 (32.4)	75 (67.6)	0.057	20 (31.7)	43 (68.3)	0.240	16 (33.3)	32 (66.7)	0.245
More than RM 10960 (T20 group)	14 (35.0)	26 (72.2)		12 (40.0)	18 (60.0)		2 (24.6)	8 (75.4)	

[1]Percentage of personal income were based on 263 respondents: Klang Valley 132 respondents; Kuching 131 respondents
[2]Percentage of household income were based on 245 respondents: Klang Valley: 123 respondents; Kuching: 122 respondents
*RM, Malaysian Ringgit; 1USD = RM 4.481 as of 29 August 2022
**Source: Household Income and Basic Amenities Survey Report 2019, Department of Statistics Malaysia
Significant difference was determined using Chi-square or Fisher Exact test

Table S2: Willingness to eat grasshoppers/other insects as food in daily life based on sociodemographic and socioeconomic characteristics

**Sociodemographic Characterises**	**Are you willing to eat grasshoppers/insects as food in your daily life? n (%)**								
	**All (n=292)**			**Klang Valley (n=144)**			**Kuching (n=148)**		
	**Yes**	**No**	***p* value**	**Yes**	**No**	***p* value**	**Ya**	**Tidak**	***p* value**
**Sex**									
Male	26 (28.0)	67 (72.0)	0.003	15 (30.6)	34 (69.4)	0.009	11 (25.0)	33 (75.0)	0.122
Female	27 (13.6)	172 (86.4)		12 (12.6)	83 (87.4)		15 (14.4)	89 (85.6)	
**Age group**									
18 – 29 years old	37 (16.2)	192 (83.8)		17 (17.2)	82 (82.8)		20 (15.4)	110 (84.6)	
30 – 39 years old	10 (27.0)	27 (73.0)		8 (25.0)	24 (75.0)		2 (40.0)	3 (60.0)	
40 – 49 years old	5 (27.8)	13 (72.2)	0.226	2 (18.2)	9 (81.8)	0.736	3 (42.9)	4 (57.1)	0.090
50 years old and above	1 (18.2)	7 (87.5)		0 (0.0)	2 (100.0)		1 (16,7)	5 (83.3)	
**Religion**									
Muslim	9 (13.6)	57 (86.4)	0.279	9 (16.1)	47 (83.9)	0.511	0 (0.0)	10 (100.0)	0.210
Non-Muslim	44 (19.5)	182 (80.5)		18 (20.5)	70 (79.5)		26 (18.8)	112 (81.2)	

To be continue…

…continue

Sociodemographic Characterises	Are you willing to eat grasshoppers/insects as food in your daily life? n (%)								
	**All (n=292)**			**Klang Valley (n=144)**			**Kuching (n=148)**		
	**Yes**	**No**	***p* value**	**Yes**	**No**	***p* value**	**Yes**	**No**	***p* value**
**Highest education level**									
Primary	0(0.0)	1(100.0)		0(0.0)	0(0.0)		0(0.0)	1(100.0)	
Secondary	22(14.9)	126(85.1)	0.322	11(18.3)	49(81.7)	0.914	11(12.5)	77(87.5)	0.117
Tertiary	31(21.7)	112(78.3)		16(19.0)	68(81.0)		15(25.4)	44(74.6)	
**Occupation**									
Government/Semi-government servant	6 (25.0)	18 (75.0)		4 (26.7)	11 (73.3)		2 (22.2)	7 (77.8)	
Private worker	22 (31.4)	48 (68.6)		13 (28.9)	32 (71.1)		9 (36.0)	16 (64.0)	
Self-employed	3 (25.0)	9 (75.0)	0.001	0 (0.0)	6 (100.0)	0.059	3 (50.0)	3 (50.0)	0.006
Unpaid worker/ Unemployed/Retired	0 (0.0)	25 (100.0)		0 (0.0)	15 (100.0)		0 (0.0)	10 (100.0)	
Students	22 (13.7)	139 (86.3)		10 (15.9)	53 (84.1)		12 (12.2)	86 (87.8)	

To be continue…

…continue

Sociodemographic Characterises	Are you willing to eat grasshoppers/insects as food in your daily life? n (%)								
	All (n=292)			Klang Valley (n=144)			Kuching (n=148)		
	Yes	No	*p* value	Yes	No	*p* value	Yes	No	*p* value
**Personal income[1]**									
RM 1799 and below*	23 (13.7)	145 (86.3)		9 (13.2)	59 (86.8)		14 (14.0)	86 (86.0)	
RM 1800 – RM 2600	4 (19.0)	17 (81.0)	0.028	1 (12.5)	7 (87.5)	0.357	3 (23.1)	10 (76.9)	0.016
RM 2601 – RM 5499	15 (31.3)	33 (68.8)		9 (25.7)	26 (74.3)		6 (46.2)	7 (53.8)	
RM 5500 and above	7 (26.9)	19 (73.1)		5 (23.9)	16 (76.2)		2 (40.0)	3 (60.0)	
**Household income**[2]**									
Less than RM 4860 (B40 group)	7 (7.4)	87 (92.6)		3 (10.0)	27 (90.0)		4 (6.3)	60 (93.8)	
RM 4860 – RM 10959 (M40 group)	20 (18.0)	91 (82.0)	0.008	10 (15.9)	53 (84.1)	0.215	10 (20.8)	38 (79.2)	0.016
More than RM 10960 (T20 group)	11 (27.5)	29 (72.5)		8 (26.7)	22 (73.3)		3 (30.0)	7 (70.0)	

[1]Percentage of personal income were based on 263 respondents: Klang Valley 132 respondents; Kuching 131 respondents
[2]Percentage of household income were based on 245 respondents: Klang Valley: 123 respondents; Kuching: 122 respondents
*RM, Malaysian Ringgit; 1USD = RM 4.481 as of 29 August 2022
**Source: Household Income and Basic Amenities Survey Report 2019, Department of Statistics Malaysia
Significant difference was determined using Chi-square or Fisher Exact test

Table S3: Visual acceptability of respondents based on study location

Forms of grasshoppers/ insects[2]	Mean Rank (n=292)			
	All (n=292)	Klang Valley (n=144)	Kuching (n=148)	*p* value[1]
Roasted grasshoppers/insects	2.64[a]	2.59[a]	2.68[a]	0.380
Chocolate-coated grasshoppers/insects	2.18[b]	2.18[b]	2.18[b]	0.460
Cooked meal containing grasshoppers/insects	2.40[c]	2.43[c]	2.39[c]	0.254
Biscuit made with grasshopper/insects' flour	2.77[a]	2.80[a]	2.74[a]	0.183
***p* value**	$p<0.001^{***}$	$p<0.001^{***}$	$p<0.001^{***}$	

[a,b,c]Different letters in a column indicate significant differences ($p<0.05$) using Friedman test and Post Hoc Wilcoxon signed rank test
[1]No significant different using Mann-Whitney U test
[2]Mean rank was reported
